# Artificial intelligence’s impact on breast cancer pathology: a literature review

**DOI:** 10.1186/s13000-024-01453-w

**Published:** 2024-02-22

**Authors:** Amr Soliman, Zaibo Li, Anil V. Parwani

**Affiliations:** Department of Pathology, Ohio State University, Columbus, OH USA

**Keywords:** Artificial intelligence, Machine  learning, Digital pathology, Breast cancer

## Abstract

This review discusses the profound impact of artificial intelligence (AI) on breast cancer (BC) diagnosis and management within the field of pathology. It examines the various applications of AI across diverse aspects of BC pathology, highlighting key findings from multiple studies. Integrating AI into routine pathology practice stands to improve diagnostic accuracy, thereby contributing to reducing avoidable errors. Additionally, AI has excelled in identifying invasive breast tumors and lymph node metastasis through its capacity to process large whole-slide images adeptly. Adaptive sampling techniques and powerful convolutional neural networks mark these achievements. The evaluation of hormonal status, which is imperative for BC treatment choices, has also been enhanced by AI quantitative analysis, aiding interobserver concordance and reliability. Breast cancer grading and mitotic count evaluation also benefit from AI intervention. AI-based frameworks effectively classify breast carcinomas, even for moderately graded cases that traditional methods struggle with. Moreover, AI-assisted mitotic figures quantification surpasses manual counting in precision and sensitivity, fostering improved prognosis. The assessment of tumor-infiltrating lymphocytes in triple-negative breast cancer using AI yields insights into patient survival prognosis. Furthermore, AI-powered predictions of neoadjuvant chemotherapy response demonstrate potential for streamlining treatment strategies. Addressing limitations, such as preanalytical variables, annotation demands, and differentiation challenges, is pivotal for realizing AI’s full potential in BC pathology. Despite the existing hurdles, AI’s multifaceted contributions to BC pathology hold great promise, providing enhanced accuracy, efficiency, and standardization. Continued research and innovation are crucial for overcoming obstacles and fully harnessing AI’s transformative capabilities in breast cancer diagnosis and assessment.

**Graphical Abstract:**

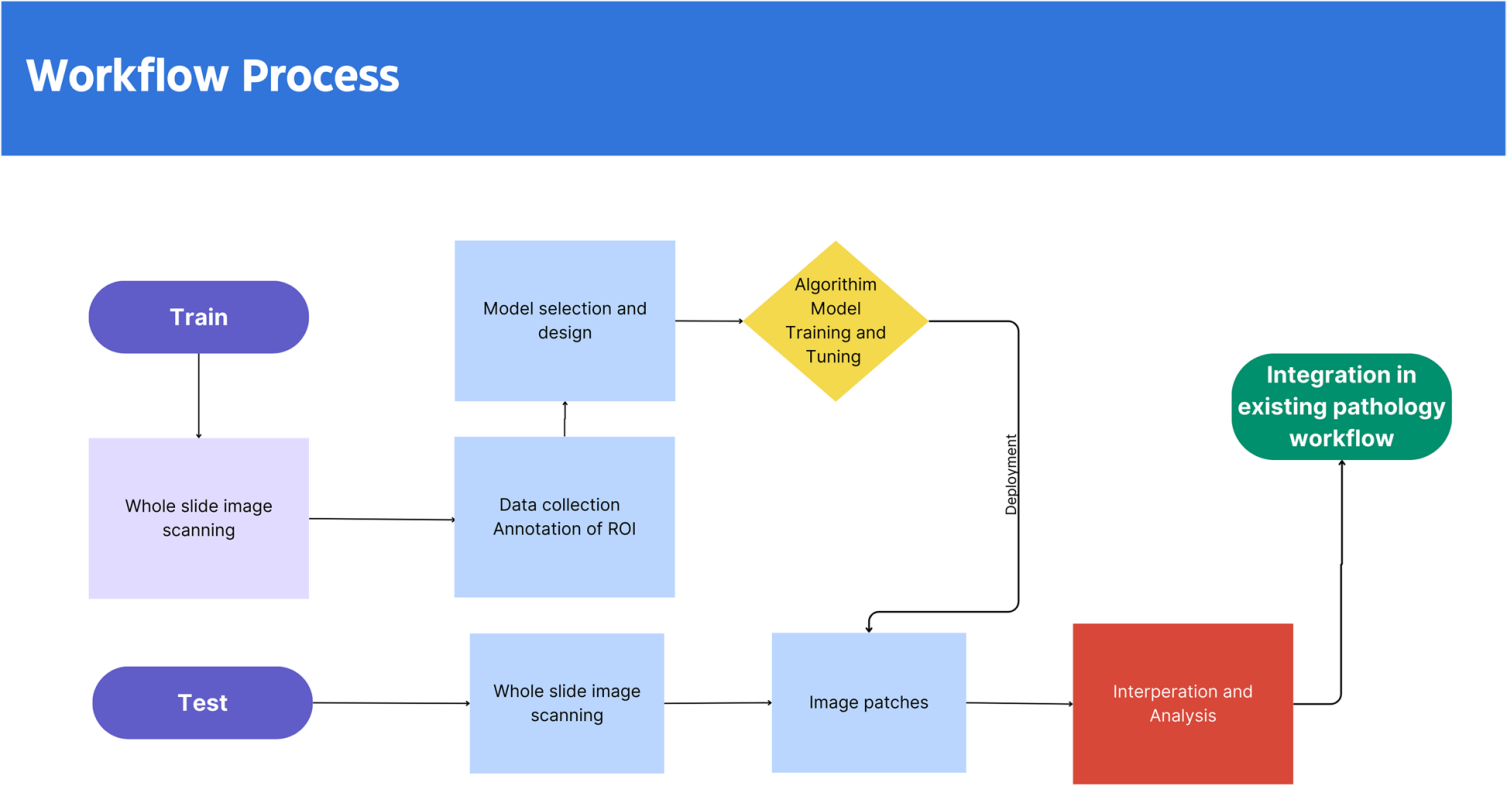

## Background

Artificial intelligence (AI) has caused a paradigm change in the world of industrial activity, and its remarkable effects are expected to spread to the field of pathology in medicine. AI is the capacity of robots to emulate or equal human intelligence (HI) [1]. Machine learning, deep learning, neural networks, natural language processing, computer vision, and cognitive computing are all key components of AI. The main goal of AI is to create technology that enables computer systems to function intelligently and independently, producing results that are on par with, if not better than, those of HI [2, 3].

Deep learning aims to enhance accuracy and minimize human error, alongside pathologists without replacing their role, fostering a collaborative approach for improved diagnostic outcomes [1]. Deep learning has emerged as a promising tool for assisting in the diagnosis and grading of breast cancer (BC), identifying lymph node metastasis, and providing prognostic and predictive information. This is significant as BC is the leading cancer type among women (over 10% in several European countries and the US) [4–6]. The use of AI algorithms in BC is also gaining popularity. Integrating these AI algorithms into the digital workflow can greatly help pathologists by making their work more efficient and accurate. It reduces repetitive tasks such as counting mitoses and detecting lymph node metastases and helps reduce diagnostic errors. Researchers have used large, high-quality datasets to develop computer programs that can detect metastasis to lymph nodes [7]. Additionally, AI algorithms can provide new tools for pathologists to handle emerging assessments. Moreover, these algorithms may even be able to replace some expensive molecular tests used in breast pathology [8]. Breast cancer treatment has evolved, with neoadjuvant chemotherapy (NAC) now commonly used, especially for locally advanced cases. With the help of AI, it has become possible to predict the effect of neoadjuvant chemotherapy [9].

However, the large variability in digital images caused by variations in preanalytical laboratory techniques, staining methods, and scanners might make subsequent image analysis difficult, which is a major factor in the lack of robustness validation for many AI systems [10–13].

### Workflow diagram

Based on the currently available literature, *Open-source* AI applications in breast pathology can be summarized into the following categories based on their purpose.

1. Diagnostic (Table 1): The reported data focus on the development and evaluation of AI-based tools for precise diagnosis in breast pathology. It utilizes histological images and computational methods to distinguish between different grades, detect invasive parts and localize metastatic spots.
2. Predictive (Table 2): It explores the use of AI to predict specific outcome or treatment response in BC patients. This includes the quantification of biomarkers including estrogen receptor (ER), progesterone receptor (PR), HER2 and Ki-67 (some of these are also prognostic factors) for targeted therapy, the response to NAC and risk categorization for adjuvant chemotherapy in ER-positive/HER2-negative breast cancer.
3. Prognostic (Table 3): This review investigates the use of AI to assess prognostic features in BC, such as tumor infiltrating lymphocytes (TILs), mitosis counting and grading levels.

**Table 1:** Summary of AI-based diagnostic algorithms in breast cancer pathology

Year of publication	Reference	Number of slides for training / validation	Pathologists review (training/validation)	Algorithm details	Algorithm endpoints/outputs	Algorithm performance
2017	Yamamoto et al [14]	11661 myoepithelial cells in 22 cases	Three pathologists	Staining > Ilastik > CellProfiler > support vector machines (SVM)	Types of breast tumors, Myoepithelial cells morphology and precise nuclear features	Accuracy 90.9%
2018	Steiner et al [15]	Training: 60-80 Validation:70	Six pathologists	LYNA, inception V3	lymph nodes metastasis detection	Sensitivity (91% vs. 83%, *P* = 0.02)
2018	Cruz-Roa et a [11]	Training: 349 Validation: 52 Testing: 195	Three expert pathologists	HASHI (High-throughput adaptive sampling for whole-slide histopathology image analysis)	invasive breast cancer detection	Dice coefficient of 76%
2018	Fondón et al [16]	Training: 30 Validation: 70 Testing: 150 + images with artefacts included	Pathologists	SVM (Support Vector Machine) classifier with a quadratic kernel	Breast carcinoma classification on biopsies	accuracy levels ranging from 61.11% to 75.8%
2022	El Agouri et al [17]	328 digital slides from 116 surgical breast specimens	One pathologist, two qualified consultant breast pathologists	CNN, (Resnet50 and Xception)	Breast cancer detection/ diagnosis	accuracy (88%), and sensitivity (95%)
2023	Wang et al [4]	400 WSIs Training: 270 Test: 129	N/A	dual magnification mining network (Two stream network) (SL-Net and PH-Net)	lymph nodes metastasis localization	0.871 FROC score with dual magnification mining network and 0.88 FROC score with high magnification network
2023	Challa et al [18]	a validation cohort with 234 SLNs and a consensus cohort with 102 SLNs)	Three pathologists	Visiopharm Integrator System (VIS) metastasis AI algorithm	Diagnosis of lymph node metastasis	sensitivity of 100%

**Table 2:** Summary of AI-based predictive algorithms in breast cancer pathology

Year of publication	Reference	Number of slides for training / validation	Pathologists review (training/validation)	Algorithm details	Algorithm endpoints/outputs	Algorithm performance
2020	Li et al [13]	153 invasive breast carcinomas	pathologists and molecular pathologists	Visiopharm HER2-CONNECT APP	Pathological NAC response prediction	HER2 DIA connectivity has the strongest association to predict PCR
2021	Bodén et al [19]	200 analyzed areas containing 200 tumor cells	three experienced breast pathologists	Human in the loop + DIA	Ki 67 proliferatio assessment	visual estimation (eyeballing) performed significantly worse than DIA alone and DIA with human-in-the-loop corrections (*P* < 0.05)
2022	Shafi et al [20]	97 invasive breast carcinomas, 73 biopsies, 24 resections	Two Pathologists	Visiopharm automated ER (DIA) algorithm	Estrogen receptor IHC assessment	Concordance (91/97, 93.8%)
2023	Abele et al [10]	204 slides	10 participant pathologist form 8 sites	Mindpeak Breast Ki-67 RoI and Mindpeak ER/PR RoI	quantifying Ki-67, estrogen receptor (ER), and progesterone receptor (PR) in breast cancer	Agreement rates: 95.8% of Ki-67 cases and 93.2% of ER/PR cases Krippendorff’s α, 0.72
2023	Shen et al [9]	Training:207 Test: 103	pathologists	CNN analysis using the ResNeXt model, SVM and RF analysis, and t-SNE analysis	NAC response	95.15% accuracy
2023	Huang et al [21]	62 HER2-positive breast cancer (HER2 +) and 64 triple-negative breast cancer (TNBC)	two pathologists	deep neural network (DeepLabV3)	NAC response	HER2 + AUC = 0.8975; TNBC AUC = 0.7674
2023	Aswolinskiy et al [22]	Training: 721 patients Validation: 126 patients	Two pathologists,research assistants	Mitosis-Detection CNN & Segmentation CNN	NAC response	AUC between 0.66 and 0.88
2023	Saednia [23]	training:144 patients with 9430 annotated tumor beds validation 63 patients with 3574 annotated tumor beds	Board-certified breast pathologists	CoAtNet & ViT models	NAC response	AUCs of 0.79, 0.81, and 0.84 and F1-scores of 86%, 87%, and 89%, respectively

**Table 3:** Summary of AI-based prognostic algorithms in breast cancer pathology

Year of publication	Reference	Number of slides for training / validation	Pathologists review (training/validation)	Algorithm details	Algorithm endpoints/outputs	Algorithm performance
2020	Pantanowitz et al [24]	320 breast invasive ductal carcinoma cases, 16,800 digital image patches from 120 WSIs/140 digital image	Ten expert pathologists and 24 readers of varying expertise	RCNN by ResNet-101	Mitotic figures counting	Accuracy with AI = 55.2% compared to manually 43.9%
2020	Chow et al [25]	93 cases of phyllodes tumor	N/A	Image Management System viewer	Phyllodes tumor mitoses counting	correlation = .794; *R*^2^ = 0.63; *P* < .001; 95% CI, 0.270–0.373
2021	Balkenhol et al [26]	94 TNBC specimens	two histopathologists	convolutional neural networks (CNN)	TILs assessment and prognostic values	Relapse free survival HR ranging between 0.777 (CD8, IM2) and 0.915 (CD3, ITS); overall survival HR varying between 0.722 (FOXP3, ITT) and 0.908 (CD3, ITA)
2022	Wang et al [6]	Training:1567Test:1262	Pathologists	deep CNN model	Categorization of NHG2 breast tumors and its risk of recurrence	increased risk for recurrence in DG2-high (HR 1.91, 95% CI 1.11–3.29, *P* = 0.019)
2022	Mantrala et al [27]	Training: 46 Test: 91	Six pathologists	Unet, DenseNet backbone, preloaded ImageNet for TF, HoVerNet, pretrained ImageNet ResNet50-Preact for NP, LinkNet with EfficientNet B4 backbone for MC	Breast cancer grading	Tubular formation (κ = 0.471 each) Nuclear pleomorphism (κ = 0.342) and was worst for mitotic count (κ = 0.233)

Subsequently, we elucidate the existing commercial platforms (Table 4) that facilitate the incorporation of artificial intelligence into breast cancer research.

**Table 4:** Summary of commercially available AI algorithms for breast cancer pathology

Platform/algorithm	Vendor	Indication	Certified/approved by	HE slides/IHC slides	Image requirement (scanner, magnification)	Workflow requirement if any	Supported samples	Output	Publications/data
Mindpeak Breast HER2 RoI	Mindpeak	breast cancer	CE-IVD	IHC slides	WSI scanner	interactive image viewer software	anti-HER2/neu clone 4B5 assay from Ventana, EP3 from Cell Marque and HercepTest (sometimes referred to as clone A0485) from Agilent Dako	HER2 score	https://uploads-ssl.webflow.com/60424989e8e0f02a922616f9/631072d2e19725a967c1735f_Mindpeak%20Breast%20HER2%20RoI%20-%20Clinical%20performance%20evaluation%20summary%20-%20APPROVED.pdf
Mindpeak Breast Ki-67 HS	Mindpeak	breast cancer	CE-IVD	IHC slides	WSI scanner	interactive image viewer software	MIB-1 pharmDx assay from Agilent Dako and the 30–9 antibody assay from Roche Diagnostics	Ki-67 score	https://uploads-ssl.webflow.com/60424989e8e0f02a922616f9/6317189c812a662bd8aa3055_Mindpeak%20Breast%20Ki-67%20HS%20-%20Clinical%20performance%20evaluation%20summary.pdf
Mindpeak Breast ER/PR	Mindpeak	breast cancer	CE-IVD	IHC slides	WSI scanner	interactive image viewer software	SP1 assay from Roche Ventana and the EP1 assay from Agilent Dako, as well as for PR through the clone 16 assay from Leica Biosystems, the clone 1E2 assay from Roche Ventana and the clone 1294 assay from Agilent Dako	ER/PR assessment	https://uploads-ssl.webflow.com/60424989e8e0f02a922616f9/631718ea00a1495c8a8dfa3b_Mindpeak%20Breast%20ER_PR%20-%20Clinical%20performance%20evaluation%20summary.pdf
Mindpeak Breast Metastasis Detection	Mindpeak	suspicious cases with breast cancer metastasis	N/A	H&E slides	N/A	Standard personal working machine. Minimum requirements: 32 bit Processor Intel Core i5 or better; 4 GB RAM	N/A	Segmentation of tissue into cancerous and benign areas	https://www.mindpeak.ai/products/mindpeak-metastasis-detection#:~:text=Mindpeaks%20Breast%20Metastasis%20Detection%20supports,metastases%20associated%20with%20breast%20adenocarcinoma
OWKIN RlapsRisk™ BC	Mindpeak	ER + /HER2- invasive carcinoma of the breast	N/A	H&E slides	WSI scanner	IMS systems or even a shared directory	N/A	determinatin the best treatement for high risk patient of early breast cancer	Garberis IJ, Gaury V, Drubay D, et al. Blind validation of an AI-based tool for predicting distant relapse from breast cancer HES stained slides. Poster presented at: European Society for Medical Oncology (ESMO); May 9th—13th 2022; Paris France
Visiopharm ER, PR, Ki-67 APP	Visiopharm	breast cancer	CE-IVD	IHC slides	WSI scanner	IMS system	staining from Agilent, Roche or Leica	ER assessment	https://visiopharm.com/app-center/app/ki-67-breast-cancer/
Visiopharm: Invasive Tumor Detection (PDS)	Visiopharm	breast cancer	CE-IVD	IHC slides	WSI scanner	N/A	p63: Roche Ventana/Zeta, CK7:Dako, CK19: AH-diagnostics/Cell Marque/Roche Ventana, combined with any biomarker APP using VirtualTripleStaining (VTS)	Automatic detection of invasive tumor	https://visiopharm.com/app-center/app/invasive-tumor-detection-vds/
Visiopharm HER2-CONNECT™	Visiopharm	breast cancer	CE-IVD	IHC slides	WSI scanner	N/A	NordiQC HER2 biomarker	HER2 scoring	https://visiopharm.com/app-center/app/her2-breast-cancer/
Visiopharm – lymph node metastasis	Visiopharm	Breast &colorectal cancer	CE-IVD	H&E slides	WSI scanner	N/A	N/A	lymph nodes metastasis detection	https://visiopharm.com/ai-assisted-metastasis-detection-in-lymph-nodes/
Visiopharm: HER2-S(D)ISH	Visiopharm	breast cancer	N/A	Immunofluroscence slides	WSI scanner	N/A	INFORM™ HER2 Dual ISH assay from Ventana/Roche	HER2-SISH assessment	https://visiopharm.com/app-center/app/her2-sish-breast-cancer/#:~:text=The%20Human%20Epidermal%20Growth%20Factor,response%20to%20HER2%2Dtargeted%20treatment
Visiopharm: HER2-FISH	Visiopharm	breast cancer	N/A	Immunofluroscence slides	WSI scanner	N/A	N/A	HER2-FISH assessment	https://visiopharm.com/app-center/app/her2-fish-breast-cancer/#:~:text=The%20Human%20Epidermal%20Growth%20Factor,response%20to%20HER2%2Dtargeted%20treatment
PathAI: AIM-HER2	PAthAI	Breast cancer	N/A	IHC slides	Leica Aperio® AT2 and GT450; Hamamatsu NanoZoomer® s360	N/A	Ventana 4B5 and Dako HercepTest™	HER2 score, Area of invasive carcinoma; Additive multiple instance learning (aMIL) density heatmap	https://pathaiwp.wpenginepowered.com/wp-content/uploads/2023/01/Copy-of-ESMO_PathAI_DS_BG_FINAL.pdf
Paige Breast Suite	Paige	Breast cancer	CE-IVD and UKCA	H&E slides	Leica Aperio AT2 and GT450 Scanners	clinical-grade viewer, FullFocus	N/A	detection of premalignant and malignant neoplasms	https://paige.ai/clinical/#
Paige Breast Lymph Node	Paige	Breast cancer	CE-IVD and UKCA	H&E slides	Leica Aperio AT2 and GT450 Scanners	clinical-grade viewer, FullFocus	N/A	lymph nodes metastasis detection	https://paige.ai/clinical/#
IBEX GALEN™ BREAST: AI-powered Pathology	IBEX	Breast cancer	CE-IVD	H&E slides	WSI scannrer	slide viewer	N/A	subtype detection&grading	https://ibex-ai.com/wp-content/uploads/2022/12/Sandbank_et_al-2022-npj_Breast_Cancer.pdf

#### AI in breast cancer diagnosis

Accurate histopathological diagnosis is crucial for BC as patient numbers surge and pathologist resources dwindle [3]. Therefore, efforts have been made to overcome this concern. Fondón et al. [16] presents a computer-aided diagnosis (CAD) tool for automated malignancy assessment of breast tissue samples. The method utilizes histological images and calculates three sets of features related to nuclei, color regions, and textures. The method was evaluated rigorously using cross-validation and external image sets, achieving accuracy levels ranging from 61.11% to 75.8%. The results demonstrate the tool’s capability to accurately distinguish between four malignancy levels (normal, benign, in situ, and malignant) and outperform other state-of-the-art methods based on feature extraction. This approach has the potential to enhance the CAD of BC and improve early diagnosis, contributing to the prevention of avoidable deaths. Another study, conducted by Cruz-Roa et al. [11] introduce a method called high-throughput adaptive sampling for whole-slide histopathology image analysis (HASHI). Traditional convolutional neural networks (CNNs) struggle with large WSIs due to the enormous number of parameters required. HASHI addresses this issue by using an efficient adaptive sampling method and a powerful CNN-based classifier. The method was trained and validated on three different data cohorts involving nearly 500 cases and independently tested on 195 studies from The Cancer Genome Atlas. The results showed that the adaptive sampling method effectively handles WSI, achieving comparable accuracy with fewer samples. The HASHI demonstrated effectiveness and robustness across different sites, scanners, and platforms, with an average Dice coefficient of 76% on an independent test dataset**.** The efforts have even been extended to include a study performed in the Middle East, specifically in Morocco, by El Agouri et al. [17]. This study aims to develop a deep learning-based approach for the efficient classification of BC histopathological images. Despite the limited data size used in this study, the classification model demonstrated good generalization performance, high accuracy, and sensitivity in detecting carcinoma rates, indicating the potential of the model to assist the pathologist in precise BC diagnosis.

The transition from the preinvasive stage of ductal carcinoma in situ (DCIS) to invasive ductal carcinoma (IDC) is a pivotal event in breast cancer progression [28]. Myoepithelial cells play a crucial role in the clinical diagnosis of DCIS and IDC. Their presence serves as a significant diagnostic criterion for pathologists. Additionally, when evaluating fine needle aspiration (FNA) cytology smears, the number of myoepithelial cells aids in differentiating between benign proliferative breast disease and invasive tumors [29]. A study conducted by Yamamoto et al. [14]. demonstrated that machine learning systems can classify different histological types of intraductal proliferative lesions based solely on subtle morphological variations observed in myoepithelial cells that cannot be detected by the human eye and without considering epithelial tumor cells. This study reveals that myoepithelial cell nuclei in DCIS lesions exhibit a distinct flattened shape that can be recognized through computational methods. Additionally, a biological mechanism based on paracrine cross-talk mechanism has been proposed to explain the progression of DCIS to IDC. This highlights the potential of using machine learning algorithms to enhance our understanding of BC pathology and improve diagnostic accuracy in identifying specific subtypes of intraductal proliferative lesions.

Metastasis location is crucial in BC staging, for which patch-based frameworks are commonly used. These frameworks extract fixed-size patches from whole slide images to train a CNN classifier that detects tumor regions. Histopathologists analyze whole slide images to identify tumor features and metastasis regions, but this process is time-consuming and prone to errors [30]. Additionally, efficiency is a challenge due to overlapping regions, leading to computational redundancy. Wang et al. [4] addressed this challenge by introducing a new two-stream network for identifying tumor metastasis cells in whole slide images. Scan-based frameworks utilize fully CNNs for faster inference by using arbitrary image blocks. Multiresolution image pyramids, combining multiple magnification patches, can improve performance and promise in BC metastasis location tasks and achieve impressive results, with a high FROC score. This method also indicates a faster inference time compared to existing approaches. Moreover, the study demonstrated the effectiveness of the high magnification network, which also contributed to the high FROC score. A recent application, by Challa et al. [18] proposes the use of a digital imaging analysis “metastasis AI” detection app (Visiopharm Integrator System metastasis AI algorithm) to screen lymph nodes for metastases in BC patients, aiming to improve diagnostic accuracy and pathologists’ efficiency. The AI algorithm demonstrated an overall sensitivity and negative predictive value (NPV) of 100%, making it a promising screening tool before pathologists’ review of H&E-stained slides. The app’s integration with the clinical workflow reduced the need for immunohistochemical staining in some cases. However, the study had limitations, including a retrospective design and the need for pathologists to carefully review and confirm positive cases identified by the AI algorithm. Steiner et al. [15] reviewed lymph node metastasis in two modes: unassisted and assisted by the algorithm. The results showed that the algorithm-assisted pathologists had higher accuracy, especially in detecting small cancer cells. The review time per image was also shorter with the algorithm’s assistance. Pathologists found it easier to classify images when using the algorithm. Overall, the study suggests that using a deep learning algorithm can improve accuracy and efficiency in digital pathology.

#### Prediction of breast cancer behavior

The Ki-67 index is used to provide information about the growth rate and aggressiveness of the tumor. However, the evaluation of Ki67 proliferation has long been a subject of uncertainty among pathologists, necessitating the development of a standardized method to assess this important factor. A study by Abele et al. [10] assessed the Mindpeak Breast Ki-67 RoI and Mindpeak ER/PR RoI for quantifying Ki-67, ER, and progesterone receptor PR in breast cancer. This tool demonstrates reliability and receives confirmation from most pathologists across a wide range of image variances and indicates the potential of AI assistance to enhance interobserver agreement and improve the reliability of immunohistochemical scoring in real-world clinical settings. However, could we depend only on AI for this analysis? In this retrospective study, by Bodén et al. [19] Ki67 areas were assessed using a human-in-the-loop digital image analysis (DIA) method, and the results were compared with visual and automatic approaches. The analysis revealed that visual estimation performed significantly worse than DIA alone and DIA with human-in-the-loop corrections, as indicated by a higher standard deviation of the error in the Ki67 index. While the addition of human-in-the-loop corrections did not improve the overall results, it proved valuable in addressing major DIA errors related to faint staining and tumor-stroma separation on an individual case basis. The study suggests that human-in-the-loop corrections primarily serve to rectify significant weaknesses in DIA applications rather than refining quantifications. A recent study by Shafi et al. [20] used Visiopharm automated ER DIA, having a fully automated digital workflow with eliminating manual intervention for ER immunohistochemistry. The process involves scanning ER IHC slides and streaming them directly into the digital platform without downloading/uploading. The results demonstrate a high concordance (93.8%) between automated DIA and pathologists’ manual scoring, making it a reliable tool for determining ER status in breast carcinoma. While there are some pitfalls identified, adjustments to the algorithm or manual annotation can address them effectively. The integration of automated DIA into routine clinical workflow is shown to be feasible, potentially saving time and labor for pathologists. Moving to HER2, this is the first study to investigate the correlation between the response to anti-HER2 NAC and HER2 protein expression using digital image analysis (Visiopharm HER2-CONNECT App) in HER2 + BC patients. The study demonstrates an excellent correlation between HER2 DIA connectivity and clinical outcomes in patients treated with anti-HER2 NAC. This DIA provides an objective and quantitative assessment of HER2 protein expression and shows potential as a predictive factor for achieving a pathological complete response (pCR). Additionally, HER2 DIA values are moderately associated with HER2 FISH copy numbers/ratios, suggesting that HER2 copy number may be more accurate in predicting HER2 protein expression than the HER2/CEP17 ratio [13]. To validate this application, one of the largest studies in this regard involved 612 invasive breast carcinomas. The data demonstrate that HER2 IHC DIA is a reliable method for measuring HER2 protein expression, with excellent concordance (87.3%) with pathologists’ manual scoring and a 16% reduction in equivocal case numbers. The study also shows that HER2 IHC DIA accurately distinguishes HER2 FISH-positive and -negative cases, although a small number of discordant cases (0.8%) were observed. The HER2 IHC connectivity value correlates better with HER2 copy number than the HER2/CEP17 ratio, suggesting that it may be a more accurate predictor of HER2 protein expression and response to anti-HER2 targeted therapy [12].

Predicting the effectiveness of NAC prior to administration is crucial to avoid unnecessary treatments. A promising approach was conducted by Shen et al. [9] This study aims to predict the effectiveness of NAC in BC patients using AI analysis of H&E images of prechemotherapy needle biopsies. A novel pipeline system has been developed, consisting of three independent models focusing on different cancer atypia characteristics. This system achieved an impressive 95.15% accuracy in predicting NAC response in a test set of 103 cases. The authors suggest that this AI system could contribute to personalized medicine in NAC therapy for breast cancer. A recent study by Aswolinskiy et al. [22] introduces interpretable computational biomarkers such as TILS, PROACTING segmentation-based biomarkers and mitotic count derived from H&E stained slides, for predicting the response to neoadjuvant chemotherapy in breast cancer. The approach contrasts with existing deep-learning models, offering greater interpretability through hypothesis-driven biomarkers. The PROACTING biomarkers show potential in predicting pathological complete response (pCR). Particularly, the TILs biomarker exhibits promising sensitivity in TNBC and Luminal B cohorts, suggesting its clinical application for distinguishing responders from non-responders and reducing overtreatment. The study emphasizes the broad applicability of computational biomarkers, especially in routine clinical practice with H&E staining, and highlights considerations for improved model applicability and TIL scoring interpretation. Another study by Saednia et al. [23] introduces a hierarchical deep learning framework for predicting breast cancer response to NAC using digital histopathological images. Unlike the previous study, this model incorporates a patch-level processing module, a tumor-level processing module, and a patient-level response prediction module, providing a comprehensive approach. The use of a self-attention-guided convolutional network based on the CoAtNet architecture at the patch level, coupled with ViT models at the tumor level, enhances the extraction of local information within tumor patches. The study emphasizes the significance of combining convolutional and self-attention modules, demonstrating superior performance in extracting informative features. Importantly, the hierarchical model outperforms two-level and patch-level-only models in predicting NAC response. The study further explores the importance of systematic strategies for fusing patch and tumor-level information on both tumor and patient levels, showcasing the necessity of a nuanced approach for more accurate predictions. Last, this recent study by Huang et al. [21] uses AI algorithms to achieve the same purpose. This approach provided robust and reproducible feature extraction, including information about the tumor immune microenvironment, such as TILs by developing of a whole slide image (WSI) feature extraction pipeline, named IMPRESS, that quantitatively evaluates histopathological features extracted from both H&E-stained and IHC-stained WSIs. Unlike previous studies, this research leverages the paired H&E and IHC images, enabling a robust and reproducible feature extraction pipeline at the WSI level. The integration of IHC-stained images provides detailed tumor immune micro-environment information, enhancing the characterization of the tumor microenvironment. The study further demonstrates the interpretability and predictive accuracy of the AI-based automatic feature extraction pipeline, outperforming models based on pathologists’ assessments and producing abundant reproducible interpretable features. Additionally, the investigation of clinicopathologic features in two breast cancer subtypes, HER2 + and TNBC, unveils common and different feature behaviors, highlighting the immunogenic nature of breast cancer.

Whitney et al. [31] investigated the use of computer analysis to predict BC risk categories determined by the Oncotype DX test. They extracted various nuclear morphology features from 178 patients’ images and used machine learning classifiers to identify the most discriminating features. The study found that computerized image analysis could potentially predict the Oncotype DX risk categories for early-stage estrogen receptor-positive breast cancer. The accuracy of the models ranged from 75 to 86% when tested on an independent validation set.

#### AI and breast cancer prognosis

AI has the potential to serve as an assisting tool for pathologists in grading breast carcinoma, contributing to a more standardized and efficient diagnosis and subsequent predictable prognosis. Breast carcinoma with a moderate grade (known as NHG 2) has been an area of concern. They are often difficult to categorize using conventional methods because they have similarities with low-grade (NGH1) and high-grade tumors (NHG3) [6]. A study conducted by Mantrala et al. [27] focused on evaluating the concordance between AI and pathologists in grading breast carcinoma. The study developed an automated framework based on deep learning to assess the Nottingham Grading System (NGS). The results revealed a moderate level of concordance for the overall grading. Importantly, there were no significant differences in concordance between pathologists alone and pathologists combined with AI. Wang et al. developed a new method called DeepGrade to classify this specific category (NHG2) more accurately. This method helps provide more consistent and precise grading. The results show that DeepGrade can also predict the prognosis of NHG2 tumors as effectively as other methods that analyze gene expression using regular stained tissue samples, which is faster and more cost-effective. DeepGrade can also provide additional information about the aggressiveness of the tumor, which can help doctors make decisions about the best treatment options, especially for certain types of BC and de-escalation of chemotherapy in particular [6].

The evaluation of mitosis in BC is important for prognosis, as it serves as a significant prognostic marker. Counting mitotic figures helps determine the grading of the tumor and aids in treatment decisions. Inter- and interlaboratory variation in manual mitotic count presents a challenge, which AI coupled with whole slide imaging aims to address. Pantanowitz et al. [24] evaluated the use of AI in quantifying mitotic figures in whole slide images of invasive breast ductal carcinoma. In this study, readers of different expertise levels then counted mitotic figures with and without AI assistance. The findings demonstrated improved accuracy, precision, and sensitivity with AI support across all experience levels while also reducing false positives and saving 27.8% of time. The study concludes that AI can enhance the precision and efficiency of mitotic figure quantification, leading to standardized pathology practices and generating enthusiasm among pathologists for integrating AI into routine tasks such as mitotic counting. Even in phyllodes tumors, accurate mitosis counting is important for grading. This study, by Chow et al. [25], aimed to determine the best method for counting mitotic figures in phyllodes tumors using digital pathology. They compared counting mitoses in 10 high-power fields (HPFs) and counting mitoses on the WSI. Both methods showed similar correlations with tumor grade, stromal atypia, and stromal hypercellularity. However, neither method showed significant correlations with patient age or tumor size.

Approximately 15% of breast cancers are classified as triple-negative, which has an aggressive nature and a high risk of recurrence [32]. Tumor-infiltrating lymphocytes are considered an important prognostic biomarker for TNBC [33, 34]. A higher density of TILs is associated with an improved prognosis [35]. Consequently, it is fundamental to assess the quantity of TILs accurately and precisely to better understand and manage TNBC. Balkenhol et al [26] explored diverse methods for objectively assessing TILs using immunohistochemically stained sections, correlating findings with patient outcomes. Employing automated deep learning analysis for TIL assessment, we scrutinized CD3, CD8, and FOXP3 markers in various tumor regions. Results revealed a consistent negative correlation between TIL abundance and recurrence-free survival (RFS) and overall survival (OS), irrespective of markers or measurement regions. Ratios between markers (CD3/CD8) were found to be poorly prognostic and discouraged. Despite the recognized prognostic value of TILs in breast cancer, a lack of standardized assessment methods hinders comparability across studies. Current protocols, such as those proposed by the International TIL Working Group (ITWG) [36], often prioritize feasibility and cost reduction. This computational pathology approach demonstrated the robustness of TILs as biomarkers, unaffected by methodological variables, suggesting potential for data-driven optimization of assessment protocols. Focusing on TNBC, exclusively assessing tumor stroma, as per ITWG guidelines, did not compromise prognostic value. The study suggests that TILs serve as reliable biomarkers, emphasizing their stability across markers and measurement regions. While influenced by the Immunoscore consortium’s methodology used in colon cancer [37], this study found both intra-tumoral and margin TILs to contain prognostic information. Combining multiple markers through ratios did not enhance prognostic value, questioning the need for more than one marker in TNBC TIL assessment. Utilizing multiplex immunohistochemistry and deep learning, the aim is to minimize human subjectivity. Despite concerns about accessibility, the study anticipates widespread AI implementation in pathology diagnostics, translating results into essays using straightforward staining protocols.

### Limitations

The integration of AI-driven solutions in BC diagnostics is transforming the landscape of pathology practices. These innovative tools empower healthcare professionals, improve accuracy, and provide standardized assessments, leading to better patient care and outcomes. As AI technology continues to advance, we can expect even more sophisticated solutions that further revolutionize BC diagnostics and treatment approaches, offering hope for a future where BC becomes more manageable and treatable.

1. Both pathologists and the AI tool encountered challenges in scoring due to restrictions stemming from various preanalytical variables. Poor sample quality, staining artifacts, the presence of air bubbles, and unfamiliar staining patterns posed difficulties in accurately assessing the samples. These factors adversely affected the performance of both human pathologists and the AI tool in scoring the samples. It highlights the importance of addressing preanalytical variables to improve the accuracy and reliability of scoring methods in pathology [10–13].
2. In terms of metastatic localization and marking numerous pixels in each image, such extensive manual annotation can be challenging and may hinder the efficiency and scalability of the algorithm. This process is labor-intensive and time-consuming [4]. In addition, the multiple-pipeline AI system to predict the efficacy of NAC relies partially on manual sampling of ROIs from annotated cancer regions [9]. Additionally, automatic annotations for ductal carcinoma (DCIS) in situ were not available through using Deep Grade method to categorize the breast NHG2 tumors and method of HASHI to detect the invasive part. This limitation could affect the accuracy of the method and to overcome it, manual annotations DCIS would be necessary [6, 11].
3. Standardization of mitosis counting and lack of agreement on area size for mitosis counting: There is a need to establish standardized methods for counting mitoses, including determining the area to count in the whole WSI. It is recommended to count multiple screens at × 40 magnification to achieve a 3 mm2 area size, equivalent to 10 high-power fields (HPF) of a standard microscope with a 0.62 field diameter [38]. The use of H&E-stained tissue sections may lead to the potential omission of mitotic figures due to tissue or imaging artifacts. The incorporation of additional biomarkers, such as Phosphorylated Histone H3 (PHH3), could have helped objectively confirm the presence of mitotic figures [24].
4. Regarding the use of this innovative crowdsourcing dataset method called scalable variational Gaussian processes (SVGPCR), the process of quantizing segmentation annotations to the patch level results in a loss of detail, which may limit its effectiveness in representing fine details in segmentation problems [39].
5. The lack of a uniform and established assessment method for TILs in BC leads to difficulty in comparing studies and a need for guidance in larger validation studies. A study, by Balkenhol et al. [26] focused specifically on TNBC, and the generalizability of the findings to other BC subtypes is unknown. The segmentation of tumor cells using automated algorithms was not optimal for some tumors, potentially introducing noise to the analysis.
6. The algorithm used in the study of Steiner et al. [15] could detect metastatic tumors but did not provide information on the positioning of these foci within the lymph nodes or specific diagnostic features. Additionally, there is increasing worry about the potential for overreliance on the algorithm, and the impact of this issue has not been investigated yet by researchers.
7. Some issues do not directly limit the use of AI in pathology. For instance, incorporating a broader range of cases can improve the representation and generalizability of AI models. Furthermore, considering additional prognostic features and investigating the influence of staining and scanning variations can help optimize the performance and applicability of AI algorithms in real-world clinical settings [31].

### Future directions

Exploring accuracy improvements in AI assistance through controlled ROI selection, investigating the impact of AI tools on diagnostic speed and interobserver variance, addressing ethical considerations, and ultimately enhancing overall accuracy for personalized patient treatment [10]. Regarding fast cancer metastasis detection, further research is needed to limit the use of pixel annotations through the usage of a two-stream network for learning histopathological images, especially in metastasis localization. This approach can enhance efficiency and facilitate faster analysis of large-scale datasets. This approach holds the potential for accelerating the identification of cancer metastases and could have broader implications for high-throughput histopathological image analysis [4]. The prospect is not limited to detection only but should extend to determine the clinical utility and value of assistive tools. By developing tools that offer detailed information about tumor foci, such as their position in relation to lymph nodes or specific diagnostic characteristics (extranodal extension and lymphovascular invasion), the accuracy of diagnoses can be improved. It is also important to assess how assistive reads impact the use of additional diagnostic tests, the categorization of prognosis, and the organization of cases based on algorithm predictions. These evaluations will provide valuable insights into the benefits of intelligent tools in digital pathology [15, 18].

In the multiple AI pipeline system to predict the NAC response, the task involves developing an AI system capable of automatically identifying and classifying specific nuclear phenotypes exhibited by different cancer subtypes. This requires implementing advanced automation techniques within the AI system, enabling it to analyze and interpret complex nuclear features from histopathological images or other relevant data sources. By achieving full automation, the AI system would streamline the process of identifying distinct nuclear phenotypes associated with various cancer subtypes, potentially leading to more accurate diagnoses and personalized treatment strategies [9].

Further research is needed to establish a standardized and data-driven approach for assessing TILs as prognostic biomarkers in breast cancer. Larger studies are required to evaluate the superiority of different image analysis algorithms in predicting survival outcomes. Exploration of additional markers and measurement regions may provide further insights into the prognostic value of TILs. The implementation of AI and digital pathology techniques, along with multiplex immunohistochemistry, may contribute to the widespread adoption of automated TIL assessment in pathology laboratories. Prospective validation studies can build upon these findings and help refine the methodology for TIL assessment, leading to improved patient outcomes and personalized treatment strategies [26].

In terms of Phyllodes Tumor Mitoses counting, further research with larger sample sizes and broader timeframes is needed to validate the findings and ensure generalizability. The development and validation of an AI algorithm for accurate mitosis counting in phyllodes tumors could potentially improve efficiency and consistency in pathology practice [25]. Currently, there is a need to establish consistent methods for counting mitotic figures in whole slide images (WSIs) used for cancer diagnosis. Determining the appropriate area to count, selecting hotspots, and deciding on the counting approach requires evidence-based studies. Unfortunately, there is no agreement on the size of the area to count using WSI, with different studies using varying sizes [40–42]. Until standardized guidelines are developed, it is recommended to count multiple screens at × 40 magnification, which would provide an area size of 3 mm2, equivalent to 10 high-power fields (HPF) of a standard microscope with a 0.62 field diameter [38].

Using Oncotype DX to integrate morphological and molecular measurements and improve BC risk assessment, particularly for intermediate-risk patients, can leverage image analysis and genomic data analysis to enhance risk stratification and treatment decisions. The study highlights the need to further explore the impact of staining and scanning variations on features and classification results, aiming for more robust AI-based pathology analysis. Future research could investigate the correlation between identified features and long-term disease recurrence or patient outcomes, establishing a direct link between AI-based risk assessment and clinical outcomes [31].

Integrating AI with human expertise can enhance the accuracy and precision of the process. Specifically, in tasks such as detecting DCIS and nonmalignant tumor presentations, manual annotation of regions of interest (ROIs) remains necessary. Human involvement ensures a comprehensive understanding of complex cases that may require expert judgment and interpretation. Thus, the collaboration between AI and human experts allows for improved outcomes in these challenging areas of diagnosis and assessment [11].

### Current commercially available AI platforms/algorithms in breast pathology

Advancements in AI are revolutionizing BC diagnostics, significantly improving accuracy, efficiency, and consistency in pathology analysis. Several innovative AI-based tools are commercially available, offering pathologists and oncologists valuable support in the fight against breast cancer.

Mindpeak (Hamburg, Germany) has made a significant contribution to BC diagnostics with its suite of automated image analysis software modules. The modules, including Mindpeak Breast HER2 ROI (region of interest, Mindpeak Breast Ki-67 HS, and Mindpeak Breast ER/PR, enable the automated analysis of digital histopathology images of human invasive breast carcinoma tissue samples without the need for manual fine-tuning in each lab. This streamlines the analysis process, making it more efficient and consistent across different institutions. Furthermore, Mindpeak’s forthcoming Metastasis Detection software is expected to aid cancer experts in detecting and visualizing metastases associated with breast cancer [10].

OWKIN (Paris, France) RlapsRisk™ BC is an innovative AI diagnostic tool designed to assist pathologists and oncologists in determining the most appropriate treatment pathway for early BC patients. The test is specifically tailored for adults with ER + /HER2- BC and has demonstrated superior cumulative sensitivity compared to standard clinical scoring systems. This tool offers new possibilities in personalized treatment planning, empowering healthcare professionals to make better-informed decisions for patients with early breast cancer.

**Fig. 1:**
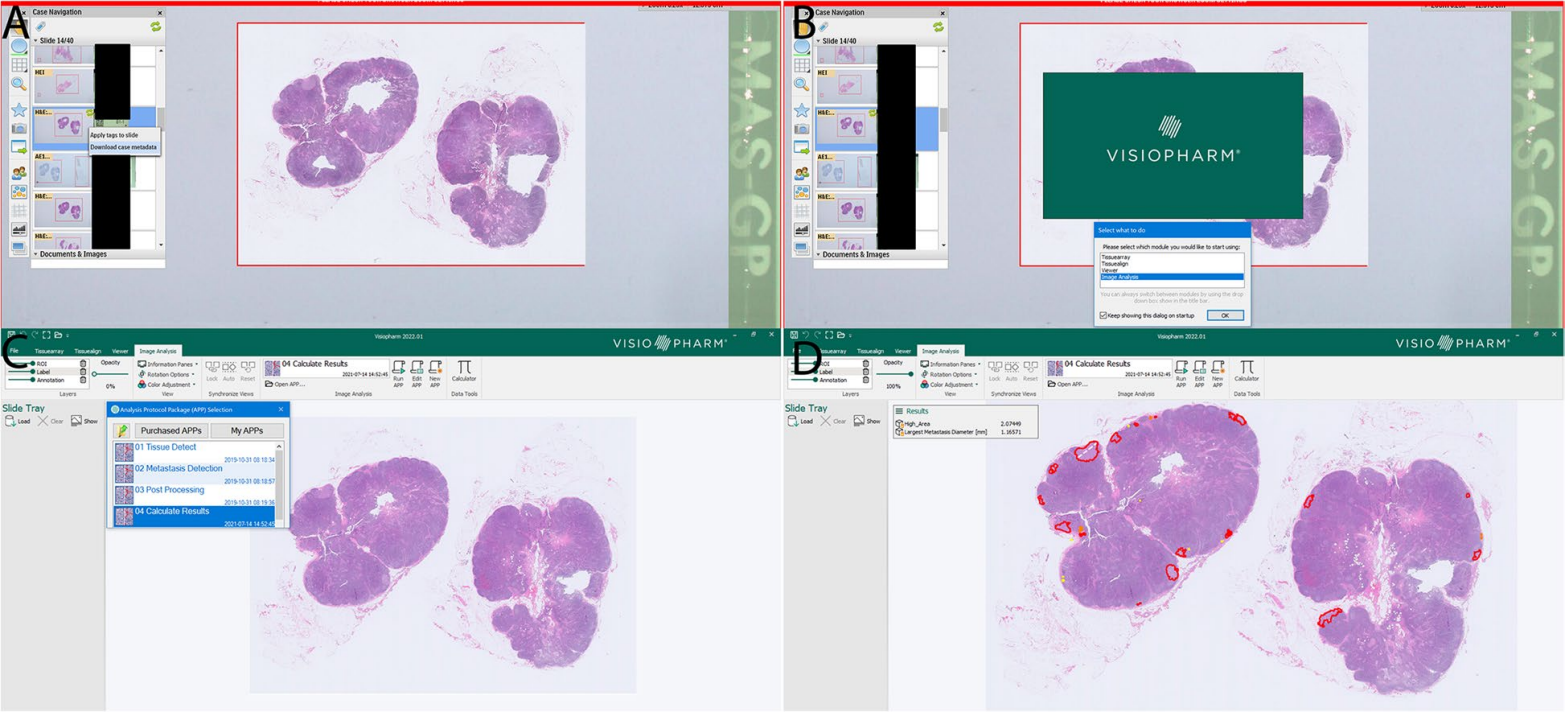
Automated lymph node metastasis detection using Visiopharm. **A** Downloading lymph node whole slide image metadata from image managing system; **B** Streaming the whole slide image in Visiopharm; **C** Analyzing lymph node metastasis using Visiopharm App; **D** demonstrating results with metastasis highlighted on image and measurement of the largest metastasis

Visiopharm, Fig. 1, (Hovedstaden, Denmark) offers a range of AI applications for BC diagnostics, certified under IVDR, ensuring adherence to rigorous regulatory standards. The Visiopharm ER APP [20] aids in determining ER positivity and negativity in BC tumors, providing primary outputs such as the percentage of positive tumor nuclei and Allred score, which is a combination of intensity and proportion score. The HER2 IHC app utilizes the HER2-CONNECT™ algorithm for automated image analysis of HER2-stained BC tissue sections, providing discrete scores corresponding to the HER2 IHC score per ASCO/CAP guidelines. Both applications streamline the analysis process and eliminate the need for manual outlining of tumor regions, saving time and increasing efficiency for pathologists [12, 13]. Visiopharm Invasive Tumor Detection (PDS) and Metastasis Detection APP: Visiopharm’s Invasive Tumor Detection (PDS) is an automated and objective method for accurately identifying invasive tumor regions. It employs a physical double stain of p63 + CK7/19 to detect invasive tumor components, excluding noninvasive components, and significantly improves workflow efficiency for pathologists. The Metastasis Detection APP simplifies lymph node assessment by accurately detecting and measuring metastases in H&E-stained lymph nodes associated with breast and colorectal adenocarcinoma. It improves sensitivity and specificity compared to manual methods and streamlines slide sorting, making it a valuable addition to the diagnostic workflow [18].

The Paige (New York, United States) Breast Suite includes Paige Breast and Paige Breast Lymph Node. Paige Breast effectively detects premalignant and malignant neoplasms, highlighting areas of cancer for confident identification. On the other hand, Paige Breast Lymph Node uses the same technology employed in detecting prostate cancer to identify metastases in lymph node slides with over 98% sensitivity. These applications enhance diagnostic confidence, prioritize cases for review, and ultimately lead to better patient outcomes. Both applications are CE-IVD and UKCA marked, ensuring adherence to the highest quality standards.

**Fig. 2:**
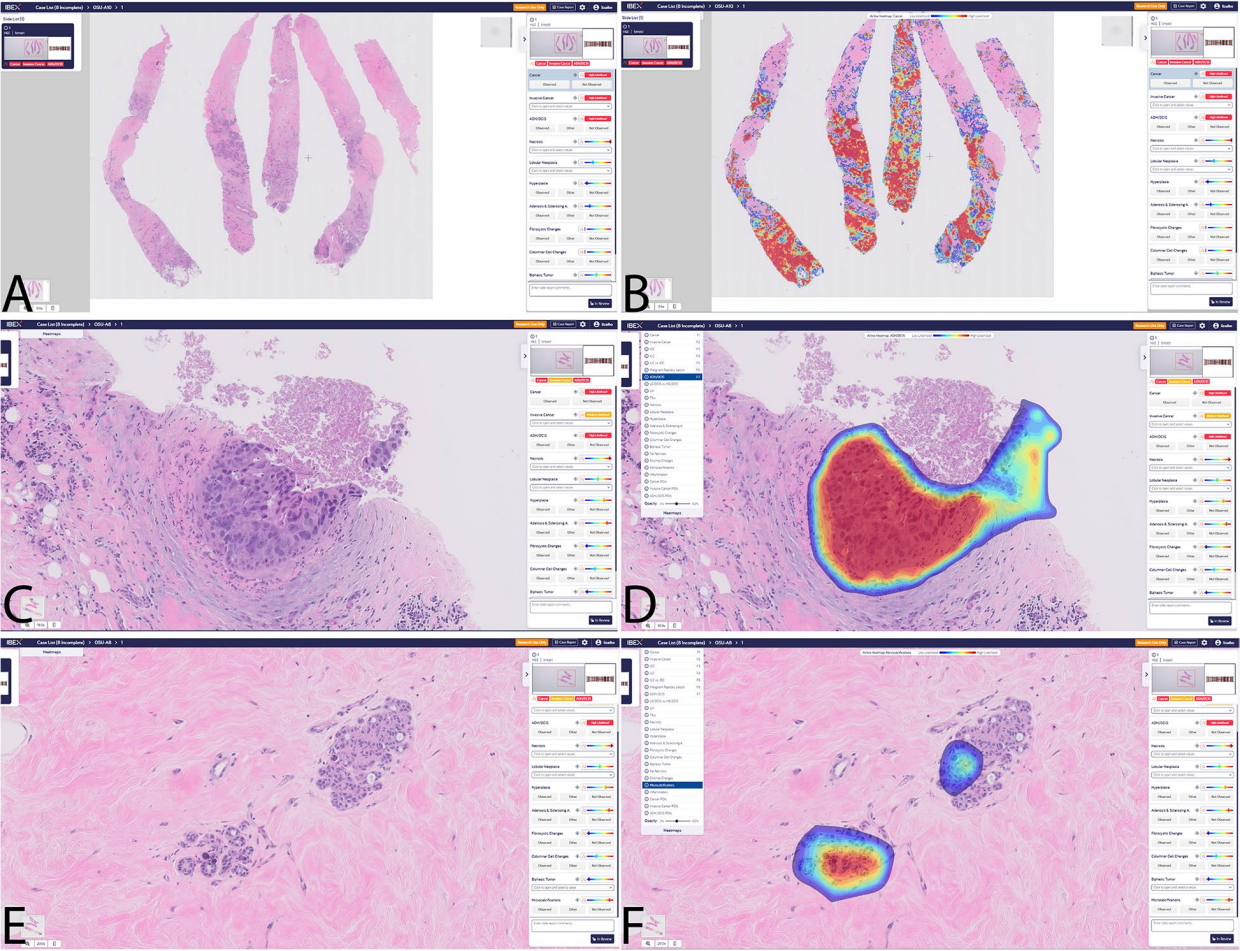
Representative images of breast lesions identified by the GALEN Breast (IBEX). **A**, **B** Invasive carcinoma; **C**, **D** Ductal carcinoma in situ; **E**, **F** Microcalcification. **A**, **C**, **E** original images; **B**, **D**, **F** Images with lesions highlighted by the GALEN Breast (heatmap)

IBEX, Fig 2, (Tel Aviv, Israel) GALEN™ BREAST is another cutting-edge AI-based diagnostic tool designed to improve productivity and shorten turnaround time for pathologists. The tool offers a wide array of features, including case prioritization, slide viewer, IHC preordering, cancer heatmaps, grading, measurements, noncancer findings, and AI-driven reporting. With its high performance in detecting invasive cancer and DCIS, IBEX GALEN™ BREAST provides unparalleled support to pathologists in accurate diagnosis and treatment planning. However, there are still limitations of AI applications for breast pathology.

The adoption of AI in pathology practice holds several expected benefits, including automation, elimination of tedious tasks, improved accuracy, and efficiency. There is significant enthusiasm among pathologists regarding the potential of AI in routine practice. Further research is needed to assess the impact of AI on patient outcomes and evaluate whether existing scoring systems need revision in light of AI’s potentially higher accuracy. Standardization efforts should be pursued, including defining equivalent units of measurement between glass slides and WSIs to ensure consistency in quantifying mitoses. The successful integration of AI into routine workflows requires continued learning and exploration of best practices. Challenges related to data quality, algorithm validation, and regulatory considerations need to be addressed to ensure reliable and ethical implementation of AI in pathology [24].

## Conclusion

In summary, this review highlights the potential of AI in assisting pathologists in various aspects of BC diagnosis and assessment. While AI has demonstrated improved accuracy, efficiency, and standardization in the realm of BC, challenges remain in addressing preanalytical variables, manual annotation requirements, and limitations in differentiating certain types of breast lesions. Further research and development are needed to overcome these limitations and fully harness the potential of AI in BC pathology.

## Data Availability

Not applicable.

## Abbreviations

HI: Human Intelligence
AI: Artificial Intelligence
BC: Breast cancer
NAC: Neoadjuvant chemotherapy
ER: Estrogen receptor
PR: Progesterone receptor
DCIS: Ductal carcinoma in situ
IDC: Invasive ductal carcinoma
CAD: Computer-aided diagnosis
CNN: Convolutional neural network
WSI: Whole Slide Image
FNA: Fine needle aspiration
DIA: Digital image analysis
pCR: Pathological complete response
NPV: Negative predictive value
ITWG: International TIL Working Group

## References

[1] Cui M, Zhang DY (2021). Artificial intelligence and computational pathology. Lab Invest.

[2] Acs B, Rantalainen M, Hartman J (2020). Artificial intelligence as the next step towards precision pathology. J Intern Med.

[3] Nam S, Chong Y, Jung CK, Kwak T-Y, Lee JY, Park J (2020). Introduction to digital pathology and computer-aided pathology. J Pathol Transl Med.

[4] Wang R, Gu Y, Zhang T, Yang J (2023). Fast cancer metastasis location based on dual magnification hard example mining network in whole-slide images. Comput Biol Med.

[5] Ferlay J, Steliarova-Foucher E, Lortet-Tieulent J, Rosso S, Coebergh JWW, Comber H (2013). Cancer incidence and mortality patterns in Europe: estimates for 40 countries in 2012. Eur J Cancer.

[6] Wang Y, Acs B, Robertson S, Liu B, Solorzano L, Wählby C (2022). Improved breast cancer histological grading using deep learning. Ann Oncol.

[7] Bándi P, Balkenhol M, van Dijk M, Kok M, van Ginneken B, van der Laak J (2023). Continual learning strategies for cancer-independent detection of lymph node metastases. Med Image Anal.

[8] Liu Y, Han D, Parwani AV, Li Z. Applications of Artificial Intelligence in Breast Pathology. Arch Pathol Lab Med. 2023;147(9):1003–13. 10.5858/arpa.2022-0457-RA 36800539

[9] Shen B, Saito A, Ueda A, Fujita K, Nagamatsu Y, Hashimoto M (2023). Development of multiple AI pipelines that predict neoadjuvant chemotherapy response of breast cancer using H&E-stained tissues. J Pathol Clin Res.

[10] Abele N, Tiemann K, Krech T, Wellmann A, Schaaf C, Länger F (2023). Noninferiority of artificial intelligence-assisted analysis of Ki-67 and estrogen/progesterone receptor in breast cancer routine diagnostics. Mod Pathol.

[11] Cruz-Roa A, Gilmore H, Basavanhally A, Feldman M, Ganesan S, Shih N (2018). High-throughput adaptive sampling for whole-slide histopathology image analysis (HASHI) via convolutional neural networks: Application to invasive breast cancer detection. PLoS One.

[12] Hartage R, Li AC, Hammond S, Parwani AV (2020). A validation study of human epidermal growth factor receptor 2 immunohistochemistry digital imaging analysis and its correlation with human epidermal growth factor receptor 2 fluorescence in situ hybridization results in breast carcinoma. J Pathol Inform.

[13] Li AC, Zhao J, Zhao C, Ma Z, Hartage R, Zhang Y (2020). Quantitative digital imaging analysis of HER2 immunohistochemistry predicts the response to anti-HER2 neoadjuvant chemotherapy in HER2-positive breast carcinoma. Breast Cancer Res Treat.

[14] Yamamoto Y, Saito A, Tateishi A, Shimojo H, Kanno H, Tsuchiya S (2017). Quantitative diagnosis of breast tumors by morphometric classification of microenvironmental myoepithelial cells using a machine learning approach. Sci Rep.

[15] Steiner DF, MacDonald R, Liu Y, Truszkowski P, Hipp JD, Gammage C (2018). Impact of deep learning assistance on the histopathologic review of lymph nodes for metastatic breast cancer. Am J Surg Pathol.

[16] Fondón I, Sarmiento A, García AI, Silvestre M, Eloy C, Polónia A (2018). Automatic classification of tissue malignancy for breast carcinoma diagnosis. Comput Biol Med.

[17] El Agouri H, Azizi M, El Attar H, El Khannoussi M, Ibrahimi A, Kabbaj R (2022). Assessment of deep learning algorithms to predict histopathological diagnosis of breast cancer: first Moroccan prospective study on a private dataset. BMC Res Notes.

[18] Challa B, Tahir M, Hu Y, Kellough D, Lujan G, Sun S (2023). Artificial intelligence-aided diagnosis of breast cancer lymph node metastasis on histologic slides in a digital workflow. Mod Pathol.

[19] Bodén ACS, Molin J, Garvin S, West RA, Lundström C, Treanor D (2021). The human-in-the-loop: an evaluation of pathologists’ interaction with artificial intelligence in clinical practice. Histopathology.

[20] Shafi S, Kellough DA, Lujan G, Satturwar S, Parwani AV, Li Z (2022). Integrating and validating automated digital imaging analysis of estrogen receptor immunohistochemistry in a fully digital workflow for clinical use. J Pathol Inform.

[21] Huang Z, Shao W, Han Z, Alkashash AM, De la Sancha C, Parwani AV (2023). Artificial intelligence reveals features associated with breast cancer neoadjuvant chemotherapy responses from multi-stain histopathologic images. NPJ Precis Oncol.

[22] Aswolinskiy W, Munari E, Horlings HM, Mulder L, Bogina G, Sanders J (2023). PROACTING: predicting pathological complete response to neoadjuvant chemotherapy in breast cancer from routine diagnostic histopathology biopsies with deep learning. Breast Cancer Res.

[23] Saednia K, Tran WT, Sadeghi-Naini A (2023). A hierarchical self-attention-guided deep learning framework to predict breast cancer response to chemotherapy using pre-treatment tumor biopsies. Med Phys.

[24] Pantanowitz L, Hartman D, Qi Y, Cho EY, Suh B, Paeng K (2020). Accuracy and efficiency of an artificial intelligence tool when counting breast mitoses. Diagn Pathol.

[25] Chow ZL, Thike AA, Li HH, Nasir NDM, Yeong JPS, Tan PH (2020). Counting mitoses with digital pathology in breast phyllodes tumors. Arch Pathol Lab Med.

[26] Balkenhol MC, Ciompi F, Świderska-Chadaj Ż, van de Loo R, Intezar M, Otte-Höller I (2021). Optimized tumour infiltrating lymphocyte assessment for triple negative breast cancer prognostics. Breast.

[27] Mantrala S, Ginter PS, Mitkari A, Joshi S, Prabhala H, Ramachandra V (2022). Concordance in breast cancer grading by artificial intelligence on whole slide images compares with a multi-institutional cohort of breast pathologists. Arch Pathol Lab Med.

[28] Sanders ME, Schuyler PA, Dupont WD, Page DL (2005). The natural history of low-grade ductal carcinoma in situ of the breast in women treated by biopsy only revealed over 30 years of long-term follow-up. Cancer.

[29] Pattari SK, Dey P, Gupta SK, Joshi K (2008). Myoepithelial cells: any role in aspiration cytology smears of breast tumors?. Cytojournal.

[30] Iqbal MJ, Javed Z, Sadia H, Qureshi IA, Irshad A, Ahmed R (2021). Clinical applications of artificial intelligence and machine learning in cancer diagnosis: looking into the future. Cancer Cell Int.

[31] Whitney J, Corredor G, Janowczyk A, Ganesan S, Doyle S, Tomaszewski J (2018). Quantitative nuclear histomorphometry predicts oncotype DX risk categories for early stage ER+ breast cancer. BMC Cancer.

[32] Voduc KD, Cheang MCU, Tyldesley S, Gelmon K, Nielsen TO, Kennecke H (2010). Breast cancer subtypes and the risk of local and regional relapse. J Clin Oncol.

[33] Dent R, Trudeau M, Pritchard KI, Hanna WM, Kahn HK, Sawka CA (2007). Triple-negative breast cancer: clinical features and patterns of recurrence. Clin Cancer Res.

[34] Pruneri G, Gray KP, Vingiani A, Viale G, Curigliano G, Criscitiello C (2016). Tumor-infiltrating lymphocytes (TILs) are a powerful prognostic marker in patients with triple-negative breast cancer enrolled in the IBCSG phase III randomized clinical trial 22–00. Breast Cancer Res Treat.

[35] Loi S, Drubay D, Adams S, Pruneri G, Francis PA, Lacroix-Triki M (2019). Tumor-infiltrating lymphocytes and prognosis: a pooled individual patient analysis of early-stage triple-negative breast cancers. J Clin Oncol.

[36] Hendry S, Salgado R, Gevaert T, Russell PA, John T, Thapa B (2017). Assessing tumor-infiltrating lymphocytes in solid tumors: a practical review for pathologists and proposal for a standardized method from the international immunooncology biomarkers working group: part 1: assessing the host immune response, TILs in invasive breast carcinoma and ductal carcinoma in situ, metastatic tumor deposits and areas for further research. Adv Anat Pathol.

[37] Pagès F, Mlecnik B, Marliot F, Bindea G, Ou F-S, Bifulco C (2018). International validation of the consensus Immunoscore for the classification of colon cancer: a prognostic and accuracy study. Lancet.

[38] Ibrahim A, Lashen A, Toss M, Mihai R, Rakha E (2022). Assessment of mitotic activity in breast cancer: revisited in the digital pathology era. J Clin Pathol.

[39] López-Pérez M, Amgad M, Morales-Álvarez P, Ruiz P, Cooper LAD, Molina R (2021). Learning from crowds in digital pathology using scalable variational Gaussian processes. Sci Rep.

[40] Al-Janabi S, van Slooten H-J, Visser M, van der Ploeg T, van Diest PJ, Jiwa M (2013). Evaluation of mitotic activity index in breast cancer using whole slide digital images. PLoS One.

[41] Roux L, Racoceanu D, Loménie N, Kulikova M, Irshad H, Klossa J (2013). Mitosis detection in breast cancer histological images An ICPR 2012 contest. J Pathol Inform.

[42] Meuten DJ, Moore FM, George JW (2016). Mitotic count and the field of view area: time to standardize. Vet Pathol.

